# Identifying Morphological Patterns of Hippocampal Atrophy in Patients With Mesial Temporal Lobe Epilepsy and Alzheimer Disease

**DOI:** 10.3389/fneur.2020.00021

**Published:** 2020-01-23

**Authors:** Yiran Duan, Yicong Lin, Dennis Rosen, Jialin Du, Liu He, Yuping Wang

**Affiliations:** ^1^Department of Neurology, Xuanwu Hospital, Capital Medical University, Beijing, China; ^2^Division of Pulmonary Medicine, Boston Children's Hospital, Boston, MA, United States; ^3^Harvard Medical School, Boston, MA, United States; ^4^Beijing Key Laboratory of Neuromodulation, Beijing, China; ^5^Center of Epilepsy, Beijing Institute for Brain Disorders, Capital Medical University, Beijing, China

**Keywords:** hippocampus, mesial temporal lobe epilepsy, Alzheimer's disease, volumetric analysis, morphologic analysis

## Abstract

**Purpose:** Mesial temporal lobe epilepsy (MTLE) and Alzheimer's disease (AD) are two distinct neurological disorders associated with hippocampal atrophy. Our goal is to analyze the morphologic patterns of hippocampal atrophy to better understand the underlying pathological and clinical characteristics of the two conditions.

**Methods:** Twenty-five patients with AD and 20 healthy controls with matched age and gender were recruited into the AD group. Twenty-three MTLE patients and 28 healthy controls with matched age and gender were recruited into the MTLE group. All subjects were scanned on 3T-MRI scanner. Automated volumetric analysis was applied to measure and compare the hippocampal volume of the two respective groups. Vertex-based morphologic analysis was applied to characterize the morphologic patterns of hippocampal atrophy within and between groups, and a correlation analysis was performed.

**Results:** Volumetric analysis revealed significantly decreased hippocampal volume in both AD and MTLE patients compared to the controls. In the patients with AD, the mean total hippocampal volume was 32.70% smaller than that of healthy controls, without a significant difference between the left and the right hippocampus (*p* < 0.05). In patients with MTLE, a significant reduction in unilateral hippocampal volume was observed, with a mean volume reduction of 28.38% as compared with healthy controls (*p* < 0.05). Vertex-based morphologic analysis revealed a generalized shrinkage of the hippocampi in AD patients, especially in bilateral medial and lateral regions. In MTLE group, atrophy was seen in the ipsilateral head, ipsilateral lateral body and slightly contralateral tail of the hippocampus (FWE-corrected, *p* < 0.05).

**Conclusions:** MTLE and AD have distinctive morphologic patterns of hippocampal atrophy, which provide new insight into the radiology-pathology correlation in these diseases.

## Introduction

Mesial temporal lobe epilepsy (MTLE) is a common type of focal epilepsy, and affects people of all ages (1). Alzheimer's disease (AD) is a neurodegenerative disorder characterized by progressive memory impairment, typically affecting people aged ≥65 years old (2). Subclinical epileptic discharges can cause significant cognitive impairment in MTLE patients (3). Therefore, a large proportion of epilepsy patients also suffer from memory dysfunction and cognitive impairment (4, 5). With the progression of dementia, the incidence of seizure ranges from 8 to 64% (6–8). Unilateral temporal epileptic discharges have also been reported in AD patients (9). Brain magnetic resonance imaging (MRI) has revealed hippocampal atrophy in both of these disorders (10–13). However, MTLE and AD are fundamentally two different disorders with distinct pathological findings. The characteristic pathologic changes in AD are the extracellular deposition of amyloid beta (Aβ) and intracellular accumulation of tau protein (14), especially in the hippocampi, with bilateral distribution (15, 16). Unilateral hippocampal sclerosis (HS) caused by hippocampal neuronal loss and gliosis is the major pathological finding of MTLE (17). Therefore, we hypothesize that the different pathological changes of the two diseases would contribute to different patterns of hippocampal atrophy.

Recent structural MRI analysis techniques, such as volumetric analysis and subfields-segmentation analysis, have been able to quantitatively assess hippocampal atrophy (18–21). However, neither volumetric measurements nor subfields segmentation can fully demonstrate the specific structural and morphological abnormalities of the hippocampus. Morphologic analysis, as a complementary tool to the volumetric analysis and subfields analysis, provides qualitative information about the specific subcortical gray matter changes in neurological disorders, which may help us to depict the detailed neuroimaging difference between the two diseases (22–24).

In this study, we applied volumetric analysis to quantitatively measure hippocampal atrophy, and morphologic analysis to qualitatively identify the patterns of hippocampal atrophy in patients with AD and MTLE. We hope to better understand the correlation between the underlying pathological process and the distinctive radiological finding of each disease.

## Materials and Methods

All procedures of the study were approved by the Ethics Committee of Xuanwu Hospital. Participants were recruited from the Department of Neurology of Xuanwu Hospital, Capital Medical University, between September 2010 and July 2017. All participants received appropriate information about the study protocol and gave written informed consent for the study and publication in accordance with the Helsinki Declaration.

### MTLE Group

Twenty-three right-handed patients with unilateral MTLE were recruited (13 males, 10 females, ages 32 ± 7.6 years). All the patients fulfilled the diagnostic criteria for MTLE according to the ILAE criteria (25): (a) clinical right-handed unilateral MTLE with typical mesial-temporal auras and ictal semiology; (b) interictal temporal spikes and/or intermittent rhythmic activities on electroencephalography (EEG); (c) unilateral HS on MRI with hippocampal atrophy on T1-weighted images, increased mesial temporal signal intensity on T2-weighted and/or fluid attenuation and inversion recovery (FLAIR)-weighted images (26); (d) no abnormal MRI findings other than HS; (e) no mass lesions (malformations of cortical development, vascular malformations, tumor); (f) no history of severe brain trauma.

Twenty-eight right-handed healthy controls with matched age and gender (13 males, 15 females, ages 28 ± 8.8 years) were recruited. Controls with any initial precipitating injury (IPI) or neurological disorder comorbidity were excluded.

Demographic and clinical data include history of initial precipitating injury (IPI) and clinical event. IPIs were defined by significant seizure and non-seizure events before age of 5, and were necessary to generate the pattern and profile of hippocampal sclerosis, including: febrile seizures, afebrile seizures, encephalitis, anoxia, head trauma, birth trauma, intracerebral bleeding (27). In our study, two of 23 patients with MTLE had febrile seizures, two patients had a history of afebrile seizures, one patient had anoxia during the perinatal period, and none of the patients had other IPIs. The clinical event showed different clinical-pathological findings of the hippocampus, including: genetic susceptibility, age of IPI, age of habitual seizure onset, latent period from IPI to habitual seizure, duration of epilepsy, frequency of epilepsy. None of our patients had family history of seizure indicating no genetic susceptibility. Our age of IPI was 4 ± 2.9, age of habitual seizure onset was 20 ± 12.6, duration of epilepsy was 12 ± 5.2, latent period was 16 ± 13.1.

### AD Group

Twenty-five patients with AD were recruited (14 males, 11 females, ages 67 ± 7.5 years). Patients were included if they had (a) Mini-Mental State Examination (MMSE) scores of 20–26, (b) Clinical Dementia Rating (CDR) score of 1, (c) Related Disorders Association criteria for AD, (d) Geriatric Depression Scale score less than or equal to 6, and (e) no other significant neurological disorder.

Twenty healthy controls with matched age and gender (12 males, 8 females, ages 64 ± 7.1 years) were recruited. Inclusion criteria were the following: no current history of depression or dementia, MMSE scores of 24 to 30 and a CDR score of zero.

### MRI Acquisition

MRI scans were obtained using a Siemens (Erlangen, Germany) 3T trio scanner. For the identification of hippocampal atrophy, the routine MR images were obtained by the following parameters. Transverse conventional T1-weighted images: repetition time (TR) = 600 ms, echo time (TE) = 8.10 ms, flip angle (FA) = 8°, field of view (FOV) = 230 mm × 180 mm, matrix = 256 × 174. Transverse turbo spin echo T2-weighted images: TR = 4,704 ms, TE = 80 ms, FA = 90°, FOV = 230 mm × 180 mm, matrix = 368 × 215. Coronal fluid-attenuated inversion recovery (FLAIR) images: TR = 9,900 ms, TE = 120 ms, FA = 120°, FOV = 230 mm × 180 mm, matrix = 276 × 136. All participants underwent the MRI scan with the same parameters. The images from patients with MTLE were visually confirmed to have HS according to the ILAE criteria, and the images from the patients with AD were visually confirmed to have bilateral hippocampal atrophy. All the MR images from healthy controls did not show any structural abnormalities.

For volumetric analysis, the T1-weighted images were acquired using a 3D magnetization prepared rapid acquisition with gradient echo (MPRAGE) sequence. The imaging parameters were: TR = 2,400 ms, TE = 3.16 ms, FA = 8°, FOV = 256 mm × 256 mm, matrix = 256 × 256, voxel size = 1 × 1 × 1 mm^3^. All the 3D T1-weighted MPRAGE images underwent automated volumetric processing.

## Analysis Procedure

### Volumetric Analysis

Volumetric measurement and comparison of the hippocampus in two groups were performed using the FreeSurfer image analysis suite (version 5.3.0, https://surfer.nmr.mgh.harvard.edu). The process included motion correction, removal of non-brain tissue by applying a hybrid watershed procedure, automated Talairach transformation, and segmentation of the subcortical gray matter volumetric structures (28, 29).

**Figure 1:**
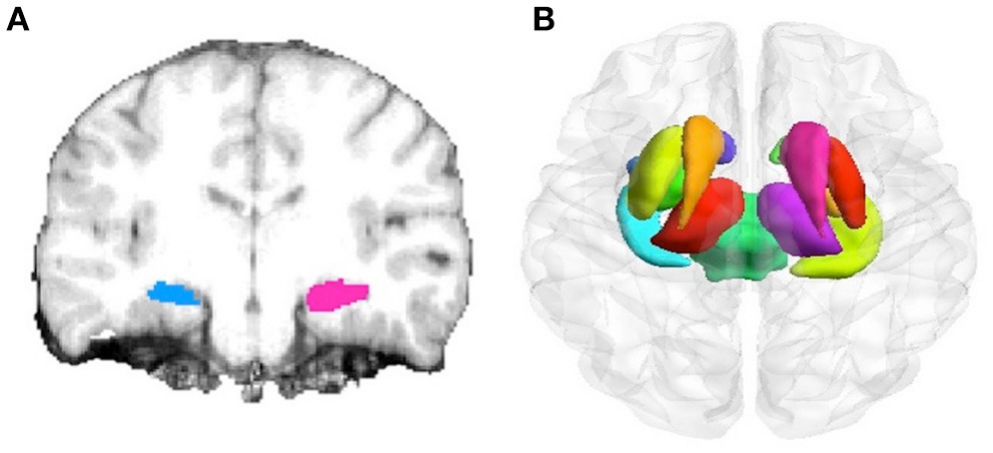
Hippocampus and subcortical structure delineation by different image segmentation tools. **(A)** Segmentation of hippocampus using the FreeSurfer surface delineation (blue) and manual delineation of the hippocampus (red) on coronal slices. **(B)** Automated segmentation of subcortical structures (including hippocampus, amygdala, caudate, nucleus accumbens, putamen, globus pallidus, and thalamus) by FSL-FIRST.

The volumetric analysis was conducted by the FreeSurfer as follows:

1. Automated segmentation and normalization. The subcortical gray matter structures underwent automated segmentation using Bayesian inference and a probabilistic atlas of hippocampal formation based on manual delineations of ultra-high resolution T1-weighted images (30). The hippocampus was defined as the region of interest (ROI), the accuracy of ROI in each subject was visually verified, and the data with segmentation errors were excluded before subsequent analyses. The most fitting hippocampal model was chosen for display (Figure 1A). Total intracranial volume (TIV) was calculated by summing the volumes of the gray matter, white matter, and cerebrospinal fluid. The raw hippocampal volume was normalized to the TIV, and to compensate for inter-individual variability in head size. Normalized hippocampal values were calculated as follows: [Raw hippocampal volume/total intracranial volume] × 1,000 (31).
2. Statistical analysis. To evaluate the hippocampal volumetric differences between groups, independent sample *t*-test and analysis were performed after adjusting for covariates (age, gender, and TIV) by a general linear model, with a priori determined significance level of *p* < 0.05 considered to be statistically significant. All data was analyzed and confirmed using the Statistical Package for Social Science Software (Version 21; IBM, Armonk, New York).

### Morphologic Analysis

#### Registration

Morphologic analysis of the hippocampus was carried out by the FSL-FIRST software (version 5.0.6, http://www.fmrib.ox.ac.uk/fsl). FSL-FIRST is a model-based registration and segmentation tool. The subcortical structures (viz. unilateral hippocampus) in volumetric T1-weighted MRI images were transformed into MNI 152 standard space to maintain the correspondence of the vertex in the following mesh models, called the registration in FIRST.

#### Standard-Flipping

In patients with MTLE, we used the standard-flipping of FSL-FISRT to quantify the differences between the right and the left portions of the hippocampi. The right hippocampal transformation of right-MTLE was coregistered to the left hippocampal transformation. The differences were parametrically tested by a minimum mean squared error and the final results were projected on the normal control hippocampus. The shapes of the ipsilateral and the contralateral hippocampi were separately compared with the normal hippocampus.

#### Automated Segmentation and Comparison

These subcortical structures were automatically segmented based on mesh models and voxel intensities. The mesh is composed of a group of triangles, and the apex of the adjoining triangles is called a vertex. The number of vertices in each mesh matched for the subcortical structure is fixed, so that corresponding vertex can be compared across subjects and between groups (i.e., MTLE group and AD group). The expected vertex correspondence is optimized by constraining within-surface motion and smoothed within the 3D deformable model (19). The shape of the model is expressed as the modes of variation (principal components), based on multivariate Gaussian assumptions (Figure 1B). After automated segmentation by FIRST, all subcortical-structure segmentations were manually double-checked to confirm the accuracy of the image registration and hippocampal segmentation. Group comparisons of vertices (control vs. MTLE, control vs. AD) were carried out using multivariate F-statistics. TIV, age, and gender are covariates of no interest (32). Statistical analysis was then performed by non-parametric permutation testing with the threshold set at cluster-level *p* < 0.05, corrected for multiple comparisons using family-wise error (FWE). The regional morphological changes of the hippocampus within the groups were explored (FWE-corrected, *p* < 0.05) (19, 33, 34).

## Results

### Volumetric Analysis

#### MTLE Group

**Figure 2:**
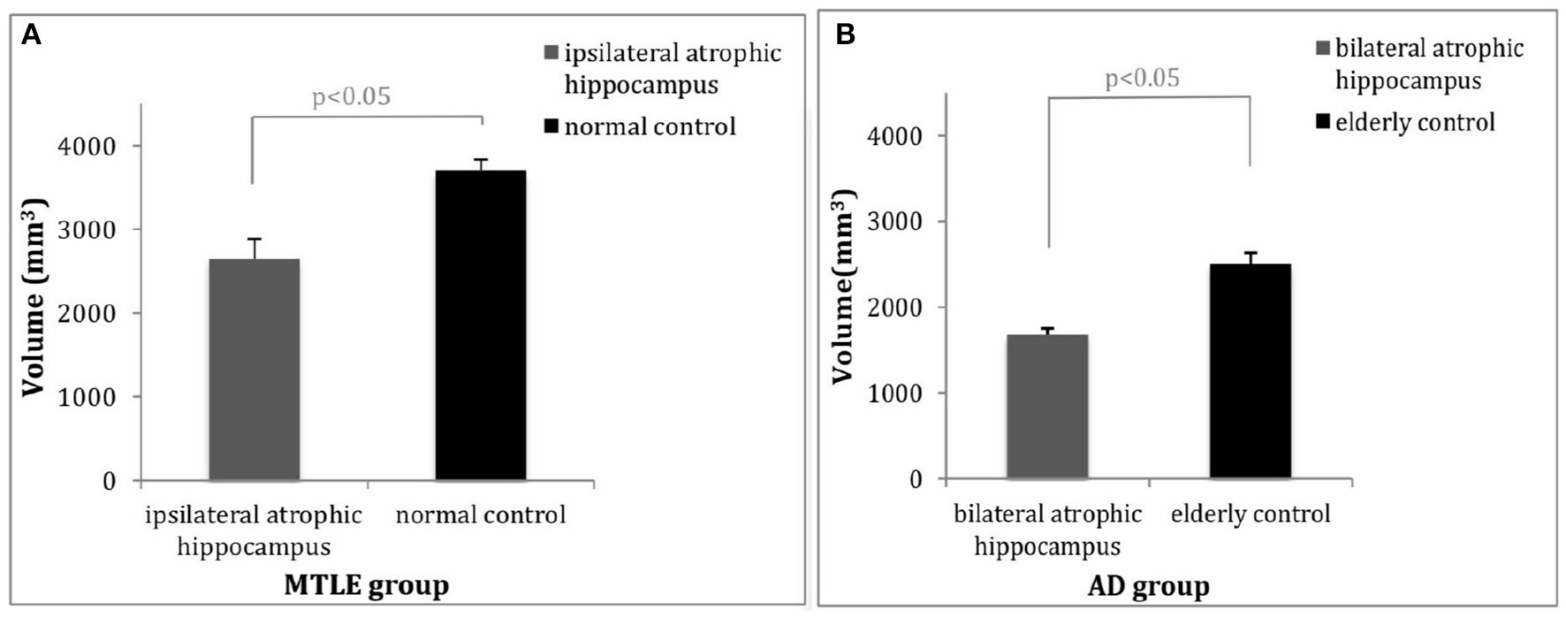
Comparisons of hippocampal volume between patients and controls. **(A)** The average volume of the ipsilateral hippocampus of the patients with MTLE was 28.38% (1,050 mm^3^) less than that of the healthy controls (*p* < 0.05). **(B)** The average volume of the hippocampi in patients with AD was 32.70% (817.44 mm^3^) less than that of the healthy controls (*p* < 0.05). MTLE, mesial temporal lobe epilepsy; AD, Alzheimer's disease.

Twenty-three MTLE patients and 28 healthy controls underwent automatic volumetric analysis of the hippocampus. After correcting for TIV, age, and gender, the unilateral hippocampal volume of the patients with MTLE was significantly reduced. The mean ipsilateral hippocampal volume of the patients was 2,650 mm^3^, which was on average 28.38% smaller as compared to the controls (the average volume of 3,700 mm^3^) (*p* = 0.015) (Figure 2A), while the mean contralateral hippocampal volume of the patients was 3624.83 mm^3^, similar to that of the control group (the average volume of 3,667 mm^3^) (*p* = 0.137).

#### AD Group

In patients with AD, significant differences in hippocampal volume were observed between the AD patients and the healthy controls after adjusting for TIV, age, and gender as covariates. The bilateral mean total hippocampal volume of patients with AD was 1682.56 mm3, whereas that of the controls was 2,500 mm^3^, 32.70% less than that of the healthy controls (*p* = 0.026) (Figure 2B). There was no significant difference observed between the volume of the left hippocampus and the right hippocampus (*p* = 0.183).

### Morphologic Analysis

#### MTLE Group

**Figure 3:**
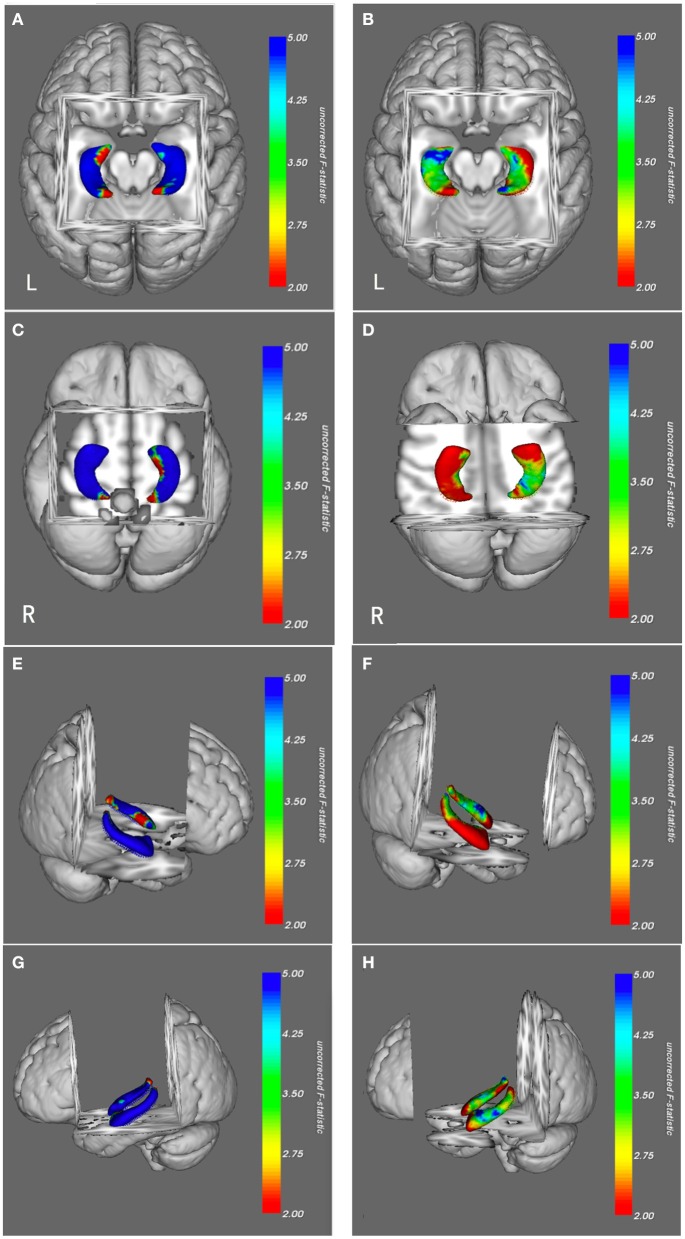
Morphologic views of hippocampi in patients with MTLE **(B,D,F,H)** and AD **(A,C,E,G)** from a superior, inferior, right lateral side and left lateral side. The results were color-coded by uncorrected F-statistic values. The transition from red to blue indicates an increase from lower to higher statistical significance, with blue indicating *p* < 0.05. Patients with MTLE had hippocampal atrophy predominantly in the ipsilateral head, partly in the ipsilateral lateral body and slightly in the contralateral tail (blue). The right-sided atrophic hippocampi of patients with MTLE were flipped to the left to facilitate comparison with those of the patients with AD. Patients with AD had generalized bilateral hippocampal atrophy, primarily in the medial and lateral regions and a small proportion of anterior and posterior regions (blue), most of the anterior and posterior regions had no significant atrophy (green and red). MTLE, mesial temporal lobe epilepsy; AD, Alzheimer's disease; FWE, Family-Wise Error.

Vertex-based morphologic analysis of the hippocampus between the patients with MTLE and the healthy controls showed significant ipsilateral (left) hippocampal atrophy in the anterior region, corresponding to the anatomical head of the hippocampus. A part of the ipsilateral lateral body of the hippocampus also had atrophy, which could be detected in Figures 3F,H (FWE-corrected, *p* < 0.05). The contralateral (right) hippocampus showed slight deformation in the posterior region, which is corresponded to the tail of the hippocampus (FWE-corrected, *p* < 0.05) (Figures 3B,D,F,H).

#### AD Group

Morphologic analysis revealed generalized deformation of bilateral hippocampi in patients with AD as compared to the healthy controls. Almost the entire hippocampi had significant atrophy in patients with AD, predominantly in the medial and lateral regions (FWE-corrected, *p* < 0.05). Although atrophy was also detected in the anterior and posterior of hippocampi, most of these regions were spared. No regional expansions were observed in comparison with the healthy controls (Figures 3A,C,E,G).

## Discussion

### Quantitative Atrophy of Hippocampus

The hippocampal atrophy in both MTLE and AD can be quantitatively measured by volumetric analysis using structural MRI techniques. We examined and measured the overall volume of the hippocampus in patients with MTLE and AD. Both showed the significant decreased volume of the hippocampus compared to their respective controls. Patients with MTLE had a 28.38% reduction of hippocampal volume unilaterally as compared with their healthy controls (*p* < 0.05), consistent with previous findings (33, 35). Patients with AD had an average 32.70% reduction of hippocampal volume bilaterally as compared with healthy controls (*p* < 0.05), without significant differences between the left and right sides. The degree of hippocampal volume loss in patients with AD was consistent with previous studies (34, 36–38). The hippocampal volume loss observed in our study confirms that the degree of hippocampal atrophy in these two diseases can be objectively and quantitatively measured by volumetric analysis.

### Distinctive Morphological Atrophy of Hippocampus

The vertex-based morphologic analysis using fully automated methods can provide qualitative information about the subcortical structures. Anatomically, the hippocampus can be divided into the head, body, and tail (22–24). On the horizontal view of a structural MRI, the head is located in the anterior region of the hippocampus, the body is in the medial and lateral regions, and the tail is in the posterior region.

The MTLE with HS is a progressive pathological injury in which clinical-pathological studies found more than one pathogenic factors. The injury factors may include IPI, genetic susceptibility, and more than one excitotoxic event occurring during or after IPI. Although IPI is a surrogate marker of cerebral injury, and some HS types are linked to specific IPI types (17), the duration of epilepsy is associated with the secondary neuron losses and hippocampal atrophy. Previous studies showed: higher prevalence of febrile convulsions [9 of 20 TLE patients (39),15 of 40 TLE patients (40)], secondary generalized tonic-clonic seizures [14 of 20 TLE patients (39)], and longer duration of epilepsy [17 ± 11 years (39), 20.0 ± 15.5 years (40)] presented extensive ipsilateral or contralateral hippocampal atrophy, some with highest effect sizes in anterior divisions. Recent studies also demonstrated that longer duration of seizures [21.3 ± 9.6 years (41), 20.8 ± 19.6 years (33), 17 ± 11 years (39)] were associated with decreased neuron densities in all hippocampal subfields and the time course to detect damage was very long (over 30 years or more). While a milder clinical course, such as a relatively shorter duration of epilepsy [14.6 ± 12.7 years (41)] was associated with marked diffused areas of hippocampal atrophy, including ipsilateral head, medioventral body, and contralateral lateral tail. In our study, the duration of epilepsy played a more important role in the pattern of hippocampal atrophy and added to different clinical-pathological findings. The patients with MTLE in our study had a shorter duration of epilepsy (12 ± 5.2 years), corresponding to the finding described above, showed more diffused hippocampal atrophies: ipsilateral head atrophy, partly ipsilateral lateral body atrophy and slightly contralateral tail atrophy.

In the AD patients, almost the entire hippocampus was atrophied, including the bilateral medial and lateral regions, while most of the anterior and posterior regions of hippocampi were spared. Cross-sectional studies showed that the hippocampus was completely atrophied in patients with late-stage of AD, whereas the atrophy in patients with mild cognitive impairment (MCI) was mostly restricted to the anterior and adjacent medial aspects of the hippocampus (42, 43). More profound hippocampal atrophy was observed on the medial and lateral aspects of hippocampus in patients with middle-stage of AD as compared to those with MCI (44–46). The patients in our group had mild-stage of AD, with CDR scores of 1, and demonstrated significant medial and lateral atrophy of the hippocampus. This pattern matches their clinical stage and also corresponds with the known pathophysiology of AD. Tau protein deposition typically started from the medial and lateral regions of the hippocampus (47). A higher burden of tau protein deposition indicated a higher severity of hippocampal atrophy (48). The neurofibrillary tangles (NFT) primarily appeared in the medial portion of the hippocampus, and then spread generally (49). These mechanisms result in neuronal loss in the hippocampus which may explain the progressive synaptic degeneration and hippocampal circuit remodeling (50).

### Morphologic Analysis Compared to the Subfields Analysis

Subfields analysis, using advanced automated structural MRI techniques, can provide useful and qualitative information about the hippocampus. According to histopathological studies, the hippocampus is divided into different subfields: the gyrus dentate (GD), and the Cornu Ammonis (CA1, CA2, CA3, and CA4), which correspond to different arrangements and shapes of pyramidal neurons (17, 41, 51). Subfields analysis can anatomically delineate these subfields, measure the volume of each subfield and detect the most significant subfield atrophy. Subfields analysis revealed that atrophy in the CA1 subfield was more prominent in MTLE patients than in healthy controls (41, 51). The CA1 subfield was vulnerable to IPI which led to the pyramidal cell loss in MTLE with HS (52, 53). In patients with AD, subfields analysis also found the evident atrophy in CA1 subfield compared to elderly controls (36, 54, 55), and the neuronal loss in CA1 subfield had been described as being primarily caused by accumulation of tau protein (56).

It is important to note that the subfield analysis had been shown to be more sensitive with the outer subfields of the hippocampus, such as CA1 and subiculum, and less sensitive with the inner subfields, such as CA3-4 and DG (57). The subfield volume estimated by this method was larger than the actual volume confirmed by manual measurement (18). Instead, we divided the hippocampus into head, body, and tail according to well-established anatomical landmarks, and assessed HS subtypes indicated gradual feature changes, particularly concerning columnar volume. In conclusion, the morphologic analysis is more accurate to describe the external shape of the hippocampus qualitatively (36, 41).

## Limitations

Limitations of this study include its relatively small sample sizes and variable clinical characteristics within and among the groups. The patients with MTLE were variable in epilepsy duration, seizure onset age, seizure frequency, seizure intractability, and medications (33, 35). The patients with AD had variability in their dementia onset age, disease duration, APOE ε4 genotype and education years (44–46). The APOE ε4 genotypes of all the AD patients were not collected and included in our study. Better controlling for these factors in further studies may help to further define the correlations between these diseases and specific patterns of hippocampal atrophy. Also, age differences between the two groups may lead to bias in outcome. Our study focused on MTLE with HS, and the mean seizure onset age of these patients was 20, consistent with the epidemiological distribution of the MTLE with HS (52). Our study recruited AD patients with average onset age at 67. We compared the patients with MTLE and AD with respective age-matched controls. Furthermore, our retrospective analysis of hippocampal atrophy was not designed to assess changes associated with disease progression. Future longitudinal prospective studies may provide more information about the relationship between hippocampal atrophy and disease progression.

## Conclusions

The distinct hippocampal atrophy patterns between MTLE and AD, as assessed by volumetric analysis and morphologic analysis, may serve as structural identifiers and provide new insight into the correlation between radiological findings and the underlying pathologies in each.

## Data Availability Statement

All datasets generated for this study are included in the article/supplementary material.

## Ethics Statement

All procedures of the retrospective study had the approval of the Xuanwu Hospital Ethics Committee and were in accordance with the Declaration of Helsinki. Participants were recruited from the Department of Neurology of Xuanwu Hospital, Capital Medical University, between September 2010 and July 2017. The informed consent was obtained from all participants in the study.

## Author Contributions

YD collected and analyzed the clinical data, designed the study and wrote the manuscript. YL oversaw data acquisition, conceived and revised the manuscript. DR reviewed all the clinical data and critically revised the manuscript. JD and LH performed post-MRI processing and analyzed the clinical data. YW supervised the study and reviewed the manuscript.

### Conflict of Interest

The authors declare that the research was conducted in the absence of any commercial or financial relationships that could be construed as a potential conflict of interest.
